# A Review of Ion Implantation Technology for Image Sensors †

**DOI:** 10.3390/s18072358

**Published:** 2018-07-20

**Authors:** Nobukazu Teranishi, Genshu Fuse, Michiro Sugitani

**Affiliations:** 1 Laboratory of Advanced Science and Technology for Industry, University of Hyogo, Ako-gun, Hyogo 678-1205, Japan; 2 Research Institute of Electronics, Shizuoka University, Hamamatsu, Shizuoka 432-8011, Japan; 3 Sumitomo Heavy Industries Ion Technology Co., Ltd., Tokyo 141-6025, Japan; genshufuse@gmail.com (G.F.); michiro.sugitani@shi-g.com (M.S.)

**Keywords:** image sensor, ion implantation, metal contamination, damage, channeling

## Abstract

Ion implantation technology is reviewed mainly from the viewpoint of image sensors, which play a significant role in implantation technology development. Image sensors are so sensitive to metal contamination that they can detect even one metal atom per pixel. To reduce the metal contamination, the plasma shower using RF (radio frequency) plasma generation is a representative example. The electrostatic angular energy filter after the mass analyzing magnet is a highly effective method to remove energetic metal contamination. The protection layer on the silicon is needed to protect the silicon wafer against the physisorbed metals. The thickness of the protection layer should be determined by considering the knock-on depth. The damage by ion implantation also causes blemishes. It becomes larger in the following conditions if the other conditions are the same; a. higher energy; b. larger dose; c. smaller beam size (higher beam current density); d. longer ion beam irradiation time; e. larger ion mass. To reduce channeling, the most effective method is to choose proper tilt and twist angles. For P^+^ pinning layer formation, the low-energy B^+^ implantation method might have less metal contamination and damage, compared with the BF_2_^+^ method.

## 1. Introduction

Solid-state image sensor technologies have advanced drastically over the last 4 decades, and have had success in the market. The sales amount of image sensors achieved 4.2 billion pieces in 2015 mainly because of the exponential growth of mobile phone market. Image sensor applications are spreading everywhere and besides mobile phones.

During the image sensor evolution, various device technologies and process technologies have been developed. Among them, ion implantation technology is one of the most important process technologies for image sensors. From the opposite viewpoint, image sensors are a very important application for ion implantation technology development. Firstly, many ion implantation steps are applied to fabricate specific structures, such as PPD (pinned photodiode) [1,2,3], special isolation structure [4], and to tune transistors at pixels [5]. Secondly, to obtain deep PD (photodiode), high energy implantations with a precise angle control are required, together with high aspect ratio resist patterns. In addition, a precise impurity profile formation is required to achieve a good signal electron transfer in a PPD pixel. Thirdly, image sensors are very sensitive to metal contamination and crystal defects, which generate white defects (blemishes) because they have low dark current and low noise.

In this paper, ion implantation technology is reviewed mainly from a viewpoint of image sensors. First, the basics of ion implantation technology are explained in Section 2. Then, metal contamination, damage and channeling, which are important topics for image sensors, are discussed in Section 3, Section 4 and Section 5, respectively. In Section 6, the P^+^ pinning layer formation methods are compared.

## 2. Basics of Ion Implantation Technology

Historically speaking, an ion implantation process patent was submitted by W. Shockley in 1949 [6], who is one of the inventors of transistors. It was applied to mass-production line in early 1970s. Therefore, it can be said that it is a rather new process technology.

At first, ion implantations were used for threshold voltage control for MOS (Metal Oxide Semiconductor) transistors. Since then, they have been adapted for various purposes;

- Threshold voltage control.
- High density doping, such as source-drain formation.
- SIMOX (separation by implantation of oxygen) [7].

Silicon dioxide layer is formed by oxygen implantation to obtain SOI (silicon on insulator) wafer.

d. Delamination [8].

High dose hydrogen implantation forms a delamination layer, and thin silicon layer is split at temperatures above 500 °C. This phenomenon is applied to produce SOI wafers by a wafer-bonding method.

e. Proximity gettering [9].

Oxygen or carbon is implanted to form gettering sites nearby the front active layer. The reproducible gettering site formation is realized by the preciseness of ion implantation, and the proximity gettering is powerful because the gettering sites are near the front side active area.

f. Dangling bond termination [10,11].

Fluorine is implanted to terminate dangling bonds. Then, interface state GR (generation recombination) centers are reduced and leakage current is decreased. Also, 1/f noise and random telegram signal (RTS) noise are reduced.

g. Amorphous formation [12].

High dose implantation forms an amorphous layer. It suppresses the channeling effect, which will be explained later. It also helps re-crystallization and electrical activation during the annealing process after ion implantation.

h. Co-implantation [13].

The impurity diffusion is suppressed if dopant atoms are implanted together with carbon, nitrogen or fluorine atoms.

Focused ion beam (FIB) and secondary ion mass spectroscopy (SIMS) also belong to a category of ion beam technology.

Ion implantation has following notable features;

(1) The doping amount is precise enough over 5 decades from 10^11^ to 10^16^ ions/cm^2^.
(2) The doping profile or depth is controlled by the ion energy.
(3) The doping area is selected by using photo-resist patterns.
(4) Doping through a thin dielectric layer on the surface can be applied if the ion energy is appropriately selected.
(5) Various species of atoms, molecules and clusters can be implanted.
(6) Ion beams have a sputtering effect.

The features from (1) to (5) are advantages for the ion implantation technology, and metal contamination, damage and channeling are disadvantages for image sensors, which will be explained in Section 3, Section 4 and Section 5, respectively.

**Figure 1:**
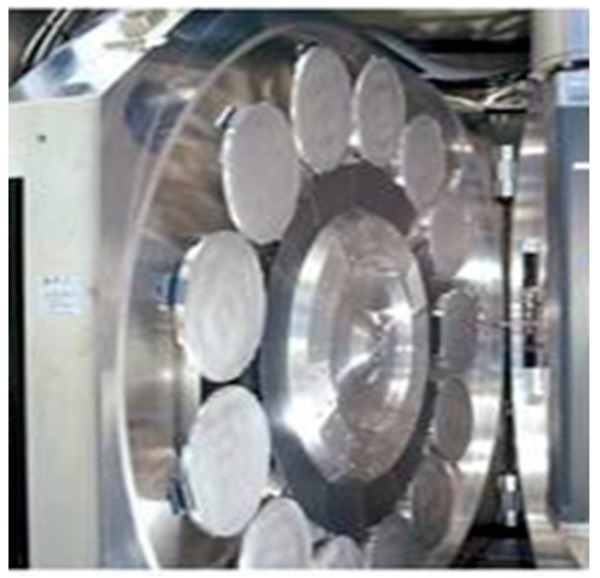
The rotating disk and wafer holders in the batch type ion implanters (courtesy of SMIT).

Ion implanters are usually classified into three categories from the viewpoint of the ion energy and beam current. The first is a medium current system mainly used for channel doping, channel stop formation and well formation. The second is a high current system mainly used for source-drain formation and contact formation. The third is a high energy system used to form deep wells and PDs. From a viewpoint of wafer setting manner, there are two categories. That is, one is a single-wafer type and the other is a batch type. In the batch type, wafers are placed on a fast-rotating disk, as seen in Figure 1, in order to disperse ion beam power on multiple wafers. The batch type is mainly applied to the high current and high energy systems because they usually generate high beam power, which is a product of beam energy by current.

**Figure 2:**
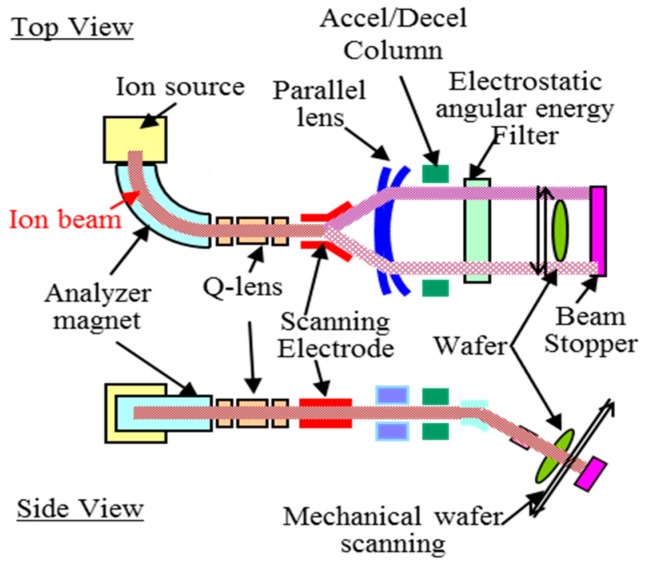
The top view and side view block diagrams of a medium current ion implanter, NV-MC3-II, of SMIT [14].

In order to explain a typical ion implanter’s structure, top view and side view block diagrams for medium-current machine, NV-MC3-II of SMIT (Sumitomo Heavy Industries Ion Technology Co., Ltd., Tokyo, Japan), are shown in Figure 2 [14]. Ions are generated from a gas or solid source material at the ion source arc block. The generated ions are extracted by the extraction electrode, to which extraction voltage is applied, and delivered to the analyzer magnet. The analyzer magnet selects ions having a desired bending radius in the magnet, *R_b_*, which is given as:

(1) $$\;R_{b}=\sqrt{2mV/qB^{2}}=p/qB$$

where *m* is ion mass, *V* is acceleration voltage, *q* is ion charge, *B* is magnetic flux density, and *p* is momentum. The Q-lens and the parallel lens shape the ion beam, and the scanning electrodes scans the ion beam to cover the entire wafer width. Next, ions are accelerated or decelerated to the needed energy at the accel/decel block if necessary. The electrostatic angular energy filter selects only the desired ions to avoid unexpected charge-exchanged ions after the analyzing magnet. Finally, they are derived into the process chamber and are implanted into the wafer. The wafer is mechanically scanned in the vertical direction perpendicular to the horizontal direction in which the ions are scanned electrically.

## 3. Metal Contamination

### 3.1. Metal Contamination for Image Sensors

**Figure 3:**
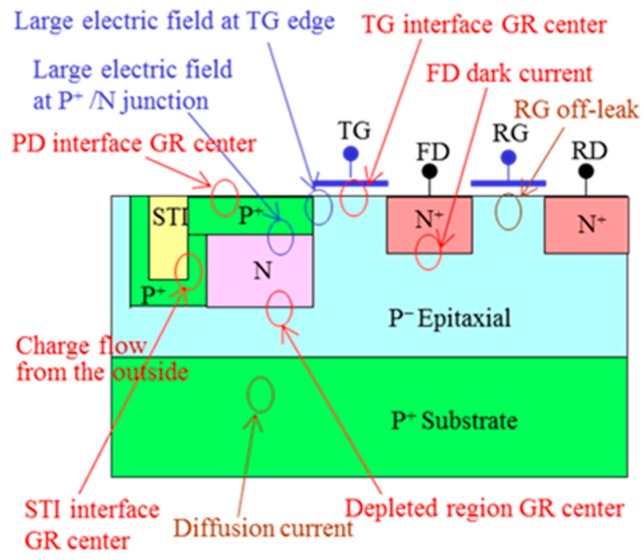
Various possible causes of dark current generation, illustrated in CMOS image sensor pixel cross-section. PD: photodiode; TG: transfer gate; FD: floating diffusion; RG: reset gate; RD: reset drain; GR center: generation recombination center; STI: shallow trench isolation.

Dark current and blemish are the most important and hardest problems facing image sensors. There are many possible causes of dark currents, which are shown in a pixel cross-section of CMOS (Complementary Metal Oxide Semiconductor) image sensor (Figure 3). One is GR centers at various locations, such as the PD interface, STI (shallow trench isolation) interface, PD depletion region, and TG (transfer gate) interface. A second is a strong electric field at the TG edge and at the junction between the P^+^ pinning layer and N PD. The others are the diffusion current from the bulk, the RG (reset gate) off-leak, and charge flow from the neighbors. Ion implantation has a possibility to generate GR centers by metal contamination and crystal damage.

Dark currents for both the neutral and depleted regions are explained by using the Shockley–Read–Hall (SRH) process. The recombination rate, *U*, is written as:

(2) $$U=\sigma v_{th}N_{t}\frac{pn-n_{i}^{2}}{n+p+2n_{i}\cosh\left(\frac{E_{t}-E_{i}}{kT}\right)}$$

where *σ* is the electron and hole capture cross section, *v_th_* is the thermal velocity, *N_t_* is the trap density, *n_i_* is the intrinsic carrier density, *E_t_* is the tarp energy level, and *E_i_* is the intrinsic Fermi level [15]. Though the trap levels at the Si–SiO_2_ interface distribute widely in the bandgap, mid-gap traps with *E_t_* = *E_i_* contribute most as (2) shows. Therefore, it is reasonable to assume that *E_t_* = *E_i_*. If depleted (*n*, *p* << *n_i_*), then, the recombination rate becomes:

(3) $$U\approx -\frac{\sigma v_{th}N_{t}n_{i}}{2\cosh\left(\frac{E_{t}-E_{i}}{kT}\right)}\approx -\frac{\sigma v_{th}N_{t}n_{i}}{2},\;$$

*U* is negative in this case and electron-hole pairs are generated. In the depleted region, generated electrons and holes drift by the electric field in the opposite directions each other. Therefore, they are not recombined, and become a dark current. One GR center generates *U*_1_:

(4) $$U_{1}=\frac{\sigma v_{th}n_{i}}{2}\;$$

Here, it is notable that the capture cross-section, *σ*, depends on metal species.

**Figure 4:**
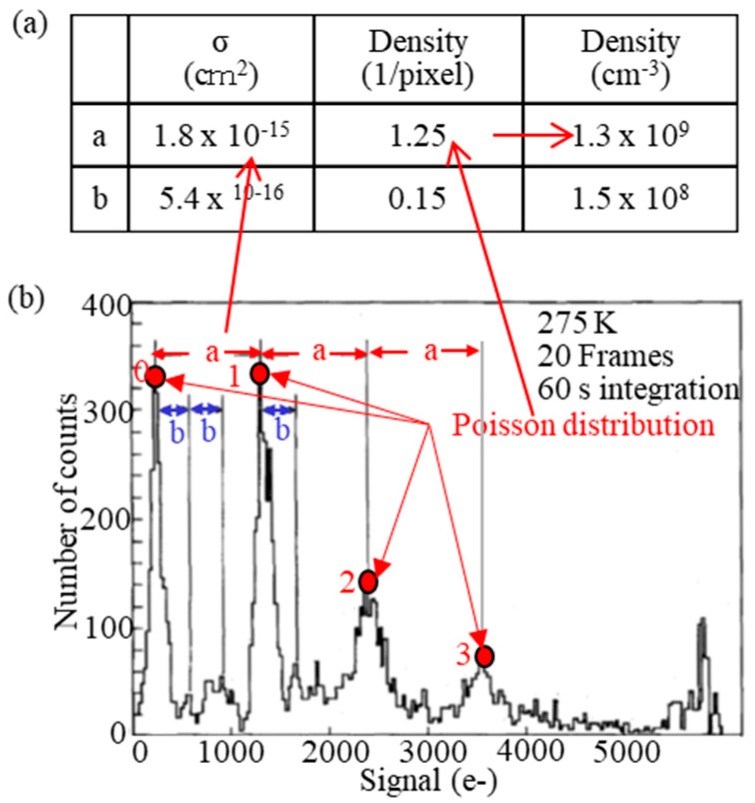
Dark current spectroscopy [16]. (**a**) Obtained capture cross sections and metal densities. (**b**) Dark current histogram, having two series of specific and periodic peaks, labeled as (**a**) and (**b**).

Figure 4 is a dark current histogram of a virtual phase CCD (Charge-Coupled Devices), which has rather many blemishes [14]. It has two series of specific and periodic peaks, labeled as “a” and “b”. Four peaks for the series “a” are seen, and they denote 0 to 3 metal atoms at a pixel from the left to the right peak. Assuming each metal is distributed as Poisson distribution, each metal’s density per pixel is derived by fitting. If the depletion region volume is estimated by the device simulation, metal density per volume can be calculated. The *σ* is derived from the peak pitch using Equation (4). The obtained metal densities and *σ*’s are shown in the inset table. The metal is identified from its cross-section. This method is called dark current spectroscopy [16]. As explained above, image sensors are so sensitive to metal contamination that even one metal atom can cause a blemish and can be detected by image sensors.

### 3.2. Metal Contamination Classification

**Figure 5:**
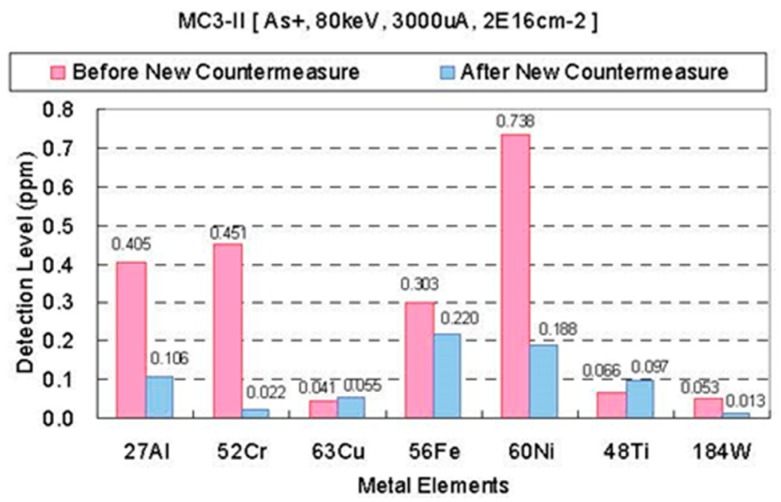
Metal contamination measurement by inductively coupled plasma mass spectrometry (ICPMS) [17]. Red denotes the metal contamination before the new countermeasure, and blue denotes that after the new countermeasure, in a MC3-II of SMIT.

Figure 5 shows examples of metal contamination measurement by ICPMS (inductively coupled plasma mass spectrometry) [17]. The samples are about 1 µm thick surface layers to which 2 × 10^16^ cm^−2^ arsenic (As) atoms are implanted with 80 keV energy. Red and blue bars denote the metal contamination before and after a new countermeasure for reduction of metal contamination is applied to the MC3-II of SMIT, respectively. Although the metal contamination is much improved by the new countermeasure, it is important to clean implanters more and in parallel to develop pixel structure and process flow, which are robust against metal contamination.

**Figure 6:**
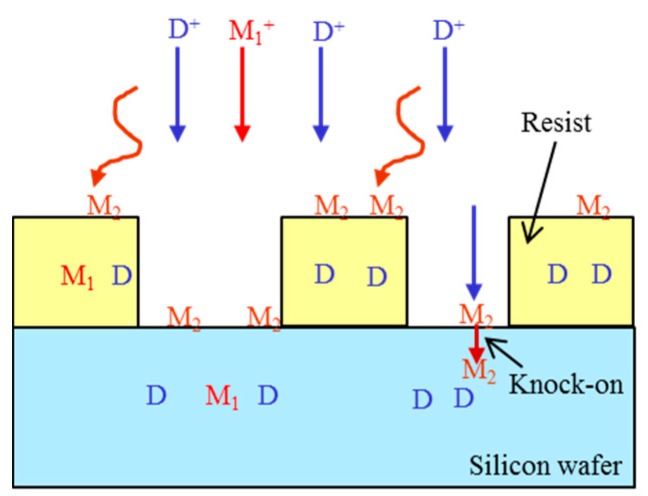
Cross-sectional illustration showing ion implantation process and metal contaminations. Straight line arrows denote energetic ion implantation, and wiggle line arrows denotes physisorption. D: dopant, M_1_: metal, which is implanted, M_2_: metal, which is physisorbed.

Metal contaminations through ion implantation are classified into two categories; one is energetic metal ion and the other is physisorption. Figure 6 shows cross sectional illustration showing ion implantation process and metal contaminations. Straight line arrows denote energetic ion implantation, and wiggle line arrows denote physisorption. Here, D is a dopant and M_1_^+^ is a metal ion, which has energy and impinges to the wafer together with the dopants. M_2_ denotes another metal atom or ion, which has a small (thermal) energy and is physisorbed on the wafer surface.

### 3.3. Physisorption Metal Contamination

**Figure 7:**
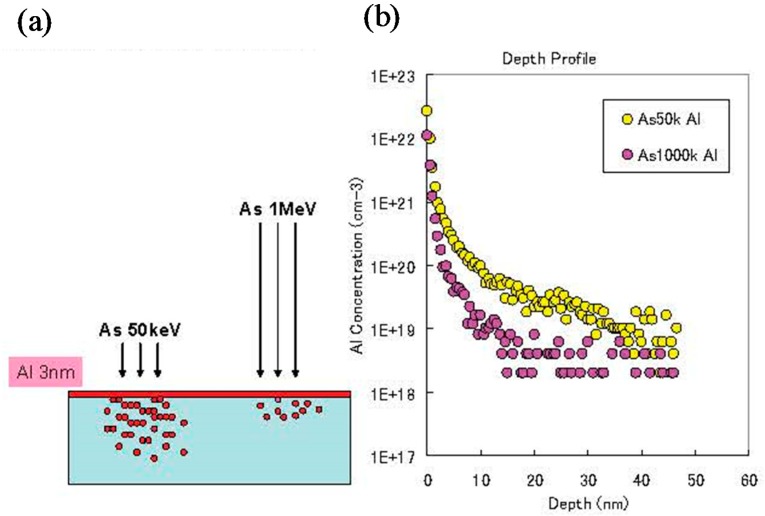
An example of knock-on effect [17]. (**a**) Illustration showing the simulation condition; (**b**) knocked-on aluminum depth profiles by Monte Carlo simulation.

Although most of the physisorbed metals are washed out by a following cleaning process, some of them invade the silicon by thermal diffusion or knock-on. To learn about the knock-on effect, Figure 7 shows the knocked-on aluminum depth profiles by Monte Carlo simulation [17]. The condition is that after a 3 nm thick aluminum layer is deposited on the silicon wafer, 1 × 10^15^ cm^−2^ As with two different energies, 50 keV and 1 MeV, is implanted. The aluminum layer emulates physisorbed metals. The aluminum depth profile becomes larger and deeper if the implantation energy is smaller. While the knocked-on aluminum atoms reach 15 nm deep in silicon by 1 MeV, they reach 45 nm deep in silicon by 50 keV. It is notable that if the ion energy is smaller, the knock-on effect becomes larger because the cross section becomes larger according to the Rutherford scattering formula.

There are two important cautions for avoiding physisorption metal contamination; (a) a protection layer, typically thin silicon dioxide layer, should be placed on the wafer during ion implantation [18]. The thickness of the protection layer should be determined by considering the knock-on depth. If the temperature during the ion implantation is high, the thermal diffusion length of metals should be considered. (b) The wafers should be cleaned up just after the ion implantations and before the thermal treatments. It is effective if the protection layer is etched even by a little amount during the cleaning. However, some knocked-on metals remain in the protection layer, and thermal treatment should be done with care for the thermal diffusion of the remained metals, or the protection layer should be removed.

### 3.4. Contamination Reduction in Implanters

To reduce metal contamination, various technologies have been developed for ion implanters. A couple of them will be explained in this subsection.

**Figure 8:**
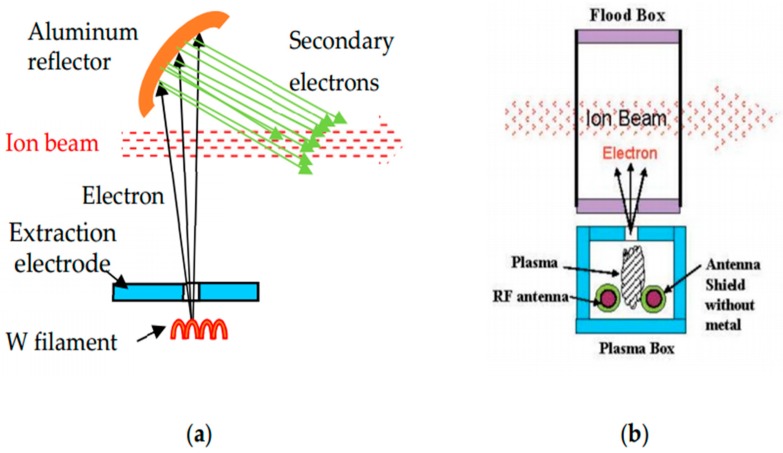
Illustrations of ion beam neutralizer evolution. (**a**) Electron shower; (**b**) plasma shower using RF (radio frequency) plasma generation [17].

One is an ion beam neutralizer, which is applied to suppress the charge up. In the early stage, an electron shower was used, which generates primary electrons by hot tungsten filament and accelerated electrons hit on an aluminum reflector to generate secondary electrons. These secondary electrons neutralize the ion beam and the wafer surface, as shown in Figure 8a. Its drawbacks are metal contamination from the tungsten filament and rather high energy of the secondary electrons for neutralization. To eliminate these drawbacks, a plasma shower was developed and has been used. As illustrated in Figure 8b [17], plasma is generated by a hot filament or radio frequency (RF) antenna in plasma box, and electrons are extracted to the flood box. Then, they neutralize the ion beam and wafer surface. Because plasma is used, the energy of the extracted electrons is small, which is good for neutralization. When RF antenna, coated with non-metal dielectric material, is used, metal contamination is greatly reduced.

Another is an energetic metal ion contamination in the case of BF_2_^+^ implantation. If the magnetic bending radius of some ion equals to that of ^11^B^19^F_2_^+^ (shortly, *R_b_* (some ion) = *R_b_* (^11^B^19^F_2_^+^)), the ion can pass through the analyzer magnet, and becomes energetic metal ion contamination. When the ion source arc chamber is made of molybdenum (Mo), Mo contamination occurs because *R_b_* (^98^Mo^++^) is exactly equal to *R_b_* (^11^B^19^F_2_^+^) [19]. Wafers are often contaminated by tungsten (W), because W is used in various parts of ion implanters, such as the ion arc chamber, filament and cathode in the ion source. The mechanism of W contamination is not as simple as the Mo case described above. Alternatively, charge exchange model for ^184^W^12^C^+^ [20] and/or molecule decomposition models for ^184^W^19^F^++^ [21] were introduced. According to the tungsten carbide (WC) charge exchange model, if a WC ion becomes double charged just before the analyzer magnet, R_b_ becomes the same as that of BF_2_. At this moment it cannot be determined which mechanism is more realistic, but at least it is true that even if arc chamber material is changed from W to carbon (C) W contamination is reduced only to half [20], which means that the effect of filament material still remains or some components other than the ion source should be considered as origins of contamination.

The electrostatic angular energy filter shown in Figure 2 after the mass analyzing magnet is a highly effective method to remove energetic metal contaminations as explained above. This is because a magnet analyzer acts as a filter of momentum per charge (mass per charge) and an electrostatic filter selects energy per charge [20]. It is notable that only part of implanters have both an analyzer magnet and electrostatic angular energy filter.

## 4. Damage

Since the energy of ion implantation is much higher than the binding energy of silicon, 4.6 eV, it generates damage (crystal defects), including vacancies, interstitials and finally an amorphous layer. After ion implantation, annealing is carried out to restore the silicon crystallinity and to activate dopants electrically. The residual defects seriously affect the following processes and device performance. One important example for effects on processes is that diffusion constants are changed due to the defects, especially vacancies and interstitials. Therefore, even if dopant profiles are the same just after the implantation, if damage is different, the final dopant profiles usually become different. Image sensors suffer from a dark current increase and blemishes caused by the damage. In this section, damage by ion implantations will be discussed.

**Figure 9:**
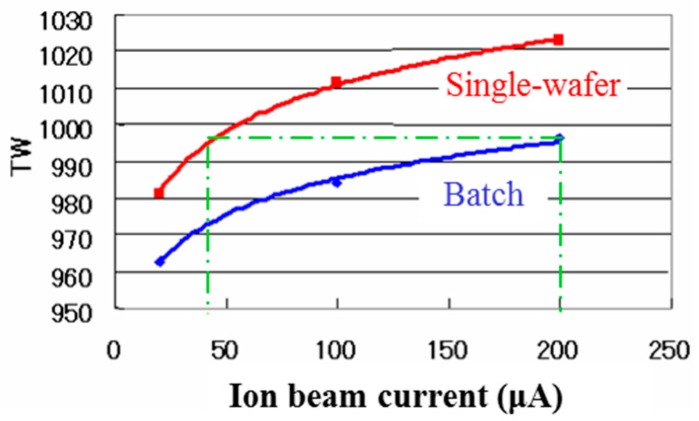
Therma wave (TW) value comparison between the single-wafer type and the batch type implanters [22]. Single-wafer type: MC3 (SMIT); batch type: GSD-HE (SMIT). Ion: P^+^, Energy: 90 keV, Dose: 2 × 10^13^ cm^−2^.

First, damage is compared between the single-wafer type, MC3 of SMIT, and the batch type, GSD-HE of SMIT, in Figure 9. To measure damage, therma wave (Therma-Wave, Inc., Fremont, CA, USA) is used, which has positive relation with the damage. The implantation condition is P^+^, 90 keV, 2 × 10^13^ cm^−2^. In GSD-HE, 13 × 200-mm-wafers are loaded at once. Both of the single-wafer type and the batch type have larger TW value or larger damage when the beam current increases in a range from 20 to 200 µA. This is reasonable result. The single-wafer type has larger TW value compared with the batch type. The TW value of the single-wafer type at 40 µA is equal to that of the batch type at 200 µA. It might be said that batch type has effectively 1/5 of the ion beam current of the single-wafer type from a damage viewpoint.

**Figure 10:**
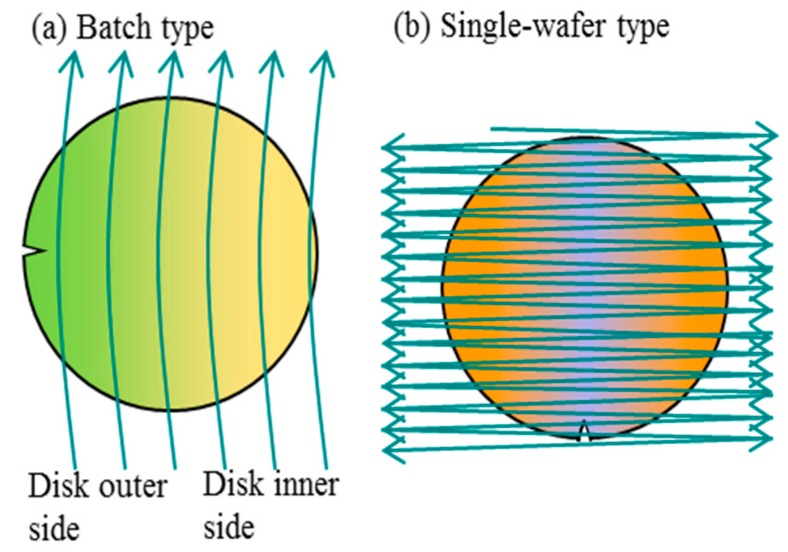
Damage distribution illustrations [17]. Arrows denote beam scans. (**a**) Batch type: the damage is smaller and more uniform. The damage at the disk inner side is slightly larger; (**b**) single-wafer type: the damage at the left side and the right side is largest.

Figure 10 illustrates the damage distribution on a wafer for the two types. The damage uniformity of the batch type is better, together with the damage level. In case of the single-wafer type, the left-hand side and the right-hand side suffer larger damage. It is because the ion beam turns back at left hand side and right-hand side, and then the beam irradiation period becomes longer and the interval becomes shorter at both sides. The batch type has a slight damage non-uniformity, where the damage at the disk inner side is larger than that at the disk outer side [17].

**Figure 11:**
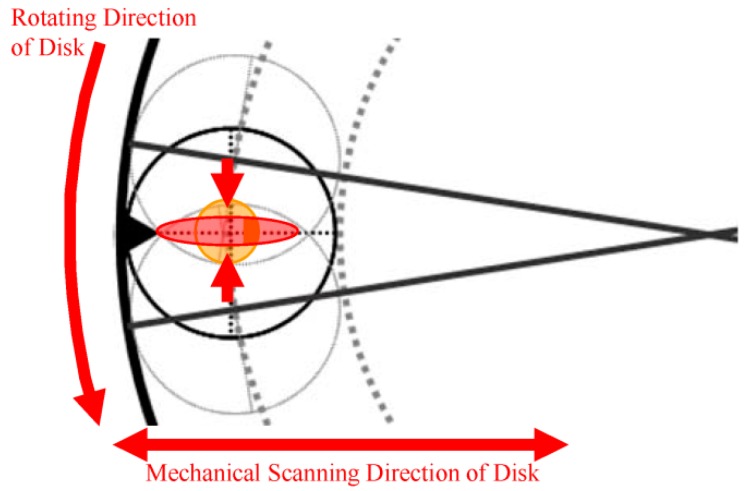
Configuration of the disk, the wafer and the beam spots [23]. Two kinds of beam spot shapes are prepared; Round shape: reference; Oval shape: to shorten the beam irradiation time.

**Figure 12:**
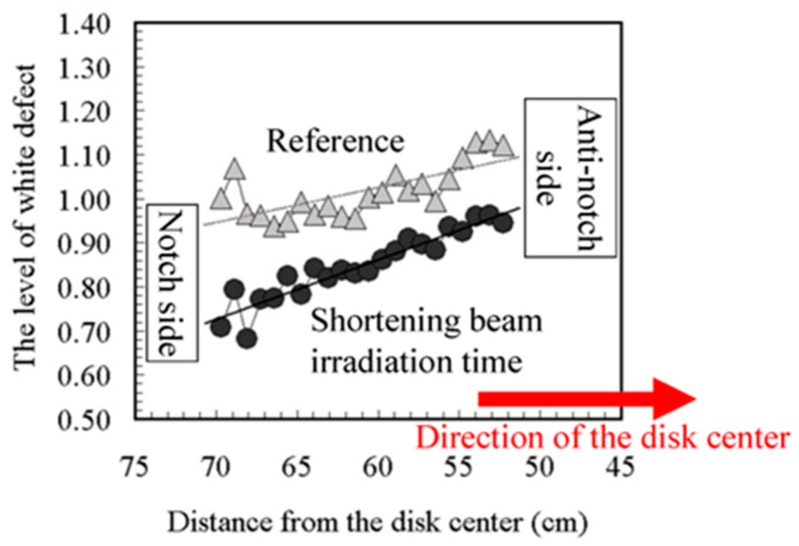
The white defect level variance on a wafer in a CCD image sensor [23].

**Figure 13:**
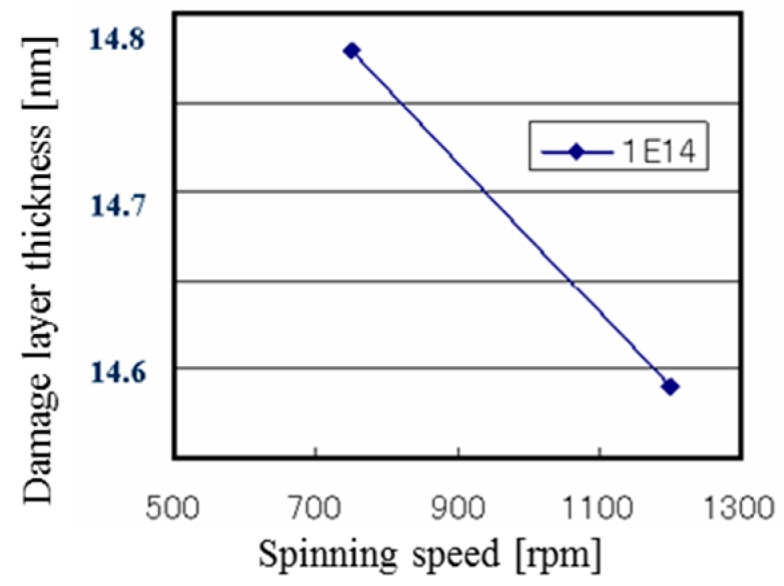
Damage layer thickness dependence on disk spinning speed [22]. Batch type: GSD-HE (SMIT); Ion: BF_2_; Energy: 20 keV; Dose: 1 × 10^14^ cm^−2^. Although amorphous layer was not generated by this experiment, a damage layer with different optical index was observed by a spectroscopic ellipsometer. The damage layer thickness indicates thickness of the damage layer.

This damage non-uniformity at the batch type is explained using another experimental result in Figure 11 and Figure 12 [23]. Figure 11 shows the configuration of the disk, the wafer and the beam spots. The distances from the disk center are 71 cm at the disk outer side, 61 cm at the wafer center, and 51 cm at the disk inner side. Since the disk spinning speed is 815 rpm, the beam moving speeds on the wafer are 6.1 cm/ms at the disk outer side, 5.2 cm/ms at the wafer center, and 4.4 cm/ms at the disk inner side. Therefore, the beam moving speed at the disk inner side is 1.4 times slower than that at the disk outer side. Two kinds of beam spot shapes are prepared; one is a conventional round shape and the other is an oval shape, which shortens the beam irradiation time. Figure 13 shows the level of white defects in a CCD image sensor. The horizontal axis is the position from the disk center. The disk inner side is located at the right-hand side. The vertical axis is the relative level of white defects. This result clearly shows that the damage at the disk inner side is larger, and the damage of the oval beam shape is smaller. Another experimental result is shown in Figure 13, which is the disk spinning speed dependence on the damage layer thickness. Although an amorphous layer was not generated by this experiment, a damage layer with different optical index was observed by a spectroscopic ellipsometer. As shown in Figure 13, the damage layer is thinner, when the disk spinning speed is larger. Even if the damage becomes smaller, the disk spinning speed cannot be increased because the resist pattern breakage by particles might become more frequent. The results, as shown in Figure 9, Figure 10, Figure 11, Figure 12 and Figure 13, suggest that even if the dose amounts and beam currents are the same, shorter irradiation time case shows smaller damage.

Next, the mass effect will be discussed. Table 1 shows the amorphous layer thickness by As dimer implantation, compared with that by as monomer implantation [22]. The energy for the As dimer is set to be twice larger than that for the monomer, and the dose is set to be half of that for the monomer to keep the equivalence. The amorphous thicknesses for the dimer are larger than those for the monomer, as shown in Table 1. Another fact is that BF_2_^+^ implantation with 1 × 10^15^ cm^−2^ dose usually generates amorphous layer, while the 1 × 10^15^ cm^−2^ B^+^ implantation, accompanied by the 2 × 10^15^ cm^−2^ F^+^ implantation, dose not generate any amorphous layer [22]. Both results imply that larger ion mass causes larger damage.

It can be said that the ion implantation damage becomes larger as the following conditions if other conditions are the same:

- Higher energy;
- Larger dose;
- Higher beam current;
- dLonger ion beam irradiation time;
- Shorter ion beam irradiation interval;
- Larger ion mass.

## 5. Channeling

**Figure 14:**
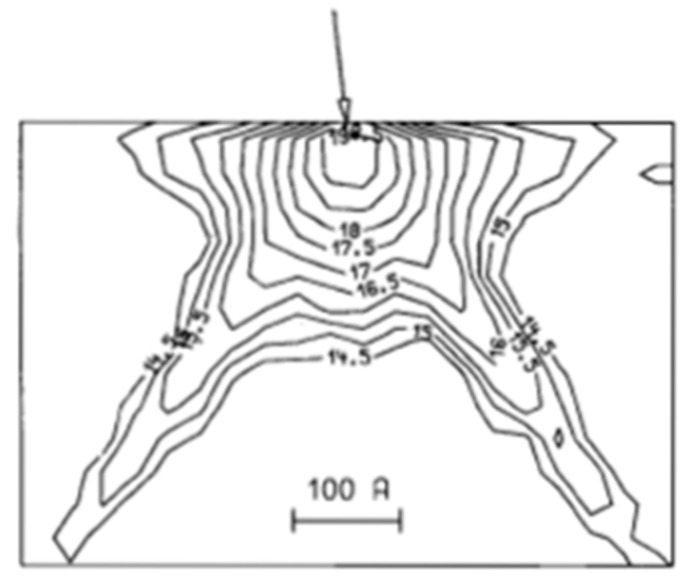
Boron concentration profile of point response by Monte Carlo simulation [25]. Wafer: (100); Ion: B^+^; Energy: 0.5 keV; Dose: 1 × 10^15^ cm^−2^; Tilt: 7°; Twist: 22°. The arrow denotes a point where B^+^ is implanted.

Because image sensors have low noise, even small irregularities are not allowed. One of the important problems is image lag in the PPD [24]. Not even a single electron should remain in the PD after the transfer period. Therefore, in order to achieve no image lag, precise design and process technology should be applied to form PPD pixels. As explained in Section 2, ion implantation can afford precise dose, depth and doping area. However, it has a large limitation, i.e., channeling. Figure 14 shows the boron concentration profile by Monte Carlo simulation as an extreme example [25]. The arrow denotes the point, where B^+^ ions are implanted with the conditions; (100) silicon wafer, 0.5 keV energy, 1 × 10^15^ cm^−2^ dose, and 7° tilt, 22° twist. This combination of angles is regarded as a small channeling condition. However, because ion energy is so small and channeling becomes so large the profile is very different from those obtained by usual amorphous model simulation. Notable fingers are formed in <110> direction due to de-channeling. Channeling brings a deeper dopant profile, which causes lower sheet resistance. It also wreaks undesirable dopant distribution dependence on channeling direction, sheet resistivity non-uniformity on a wafer, lot-to-lot sheet resistivity variation. Moreover, it might bring unexpected electric field concentrations at the fingertips. In this section, channeling will be discussed.

**Figure 15:**
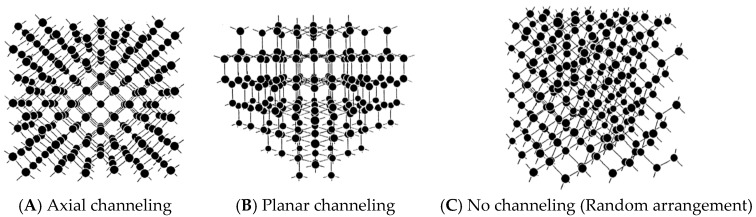
Illustrations to show the different appearance of a crystal lattice by the view angle [26].

Figure 15 illustrates the different appearance of the crystal lattice by the view angle. There are pipe-like spaces (channels) in (A) and sheet-like spaces in (B), while there are no spaces in (C), which looks like a random arrangement. If ions fly through these spaces with little collisions, it is called channeling. There are two kinds of channeling: axial channeling (A) and planar channeling (B).

**Figure 16:**
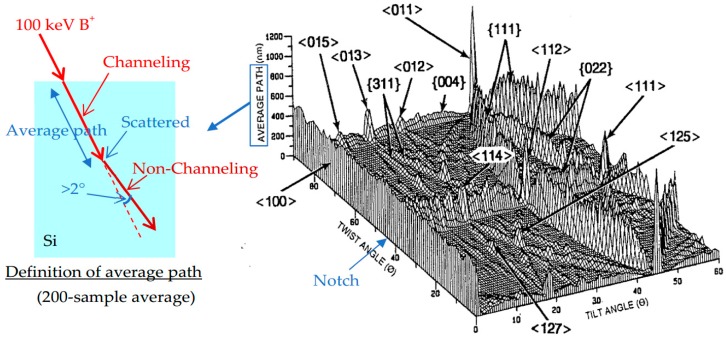
Average path dependence on the tilt and twist angles by Monte Carlo simulation [27,28,29]. The mean path is the distance along the ion trajectory until its direction deviates by more than 2° from the initial incident direction. Ion: B^+^, Energy: 100 keV, Tilt pitch: 1°, Twist pitch: 2°, Number of average: 200. The notch position is at twist =45°.

The channeling depends on the silicon surface orientation, the ion beam angle, energy, ion species, substrate temperature, etc. Figure 16 shows the average path dependence on the tilt and twist angles by Monte Carlo simulation [27,28,29]. The mean path is defined as the distance along the ion trajectory until its direction deviates by more than 2° from the initial incident direction. The average path is an average over 200 simulated paths. It can be considered as a channeling measure. The notch is located at twist =45°. The mountain ranges indicate the planer channeling, and the independent peaks are the axial channeling. There are so many axial and planer channeling directions. Among them, <011> axial is the largest. Other prominent axial channels are <112>, <100>, <111>, <013>, and <114>. Planar channels are apparent for {111}, {022}, {311}, and {004}. The preferable tilt and twist combinations to reduce channeling can be chosen by using Figure 16. If the appropriate ion implantation direction has some angle to TG to suppress the channeling, for example, multi-step implantation should be applied to keep the symmetry.

**Figure 17:**
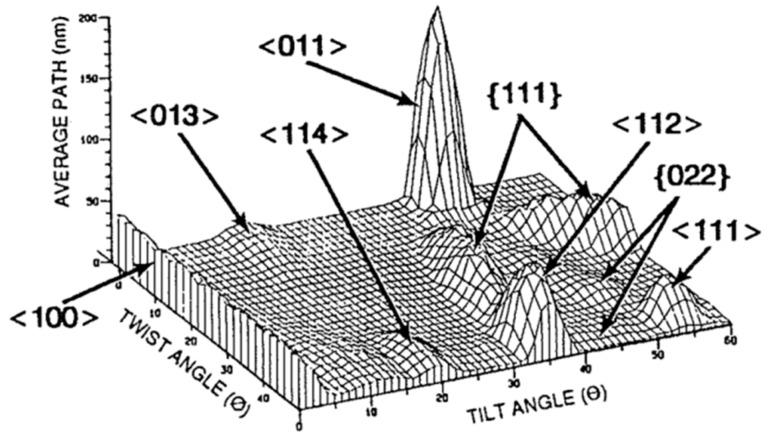
Average path dependence on the tilt and twist angles by Monte Carlo simulation [27,28,29]. Ion: B^+^; Energy: 5 keV; Tilt pitch: 1°; twist pitch: 2°.

Figure 17 shows the average path dependence on the tilt and twist angle of B^+^ with 5 keV energy. The average path becomes smaller and the peaks and mountain ranges becomes broader compared with 100 keV energy case. Because the average path is the integration from the initial to direction deviation, it becomes larger when the energy is larger. However, because the ratio of the average path to the range becomes larger when the energy becomes smaller, it can be said that the channeling is larger when the energy is smaller. Actually, the most severe problem for channeling is the tailing of the doping profile, which is determined by the final stage of the ion trajectory.

**Figure 18:**
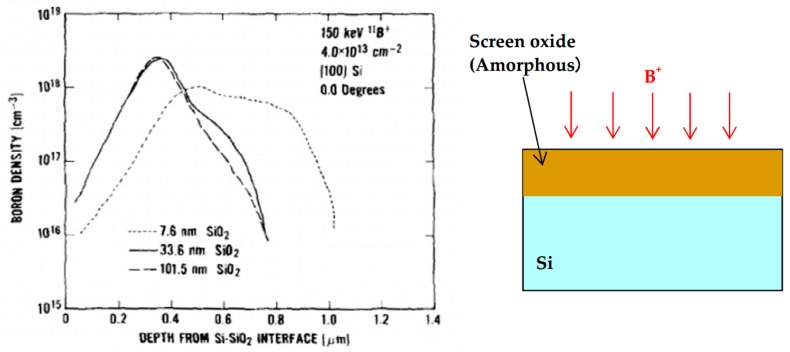
Boron density profile with various screen oxide thickness [30]. Wafer: (100) Silicon, Ion: ^11^B^+^, Energy: 150 keV, Dose: 4 × 10^13^ cm^−2^, Tilt: 0.0 ± 0.1°.

To reduce channeling, one can use the screen oxide method and amorphization method, other than beam direction selection. Figure 18 shows the effect of the screen oxide. 150 keV, 4 × 10^13^ cm^−2^, ^11^B^+^ is implanted into (100) silicon wafer with the tilt angle of 0° [30]. The screen dioxide thicknesses are 7.6 nm, 33.6 nm and 101.5 nm. Because silicon dioxide is amorphous and the ion directions are scattered, the channeling becomes smaller as the screen dioxide becomes thicker. However, in even a 100 nm oxide case, the tail by channeling still exists. Therefore, since the necessary oxide is too thick to suppress the channeling completely, the screen oxide method is not practical for the latest fine technology.

Next, the amorphization method is discussed. As explained in Section 4, the amorphous layer is generated by high dose and larger mass ion implantation. This amorphous layer reduces channeling. However, even in this case, part of ions are implanted with the channeling condition before the amorphous layer is formed. Additionally, smaller mass ions do not generate amorphous layer. In order to suppress the channeling even in these conditions, the pre-amorphization method is introduced [12]. Electrically-neutral ions, such as Ge or Si, are implanted at a high-dose beforehand to form an amorphous layer. Then, electrically-active ions, such as B, are implanted without suffering from channeling. Finally, the amorphous layer is re-crystalized by a following annealing process. This method is applied to the shallow source-drain formation for fine logic process. However, image sensors are so sensitive that there is still a room for improvement on the re-crystallization quality in this method at present.

**Figure 19:**
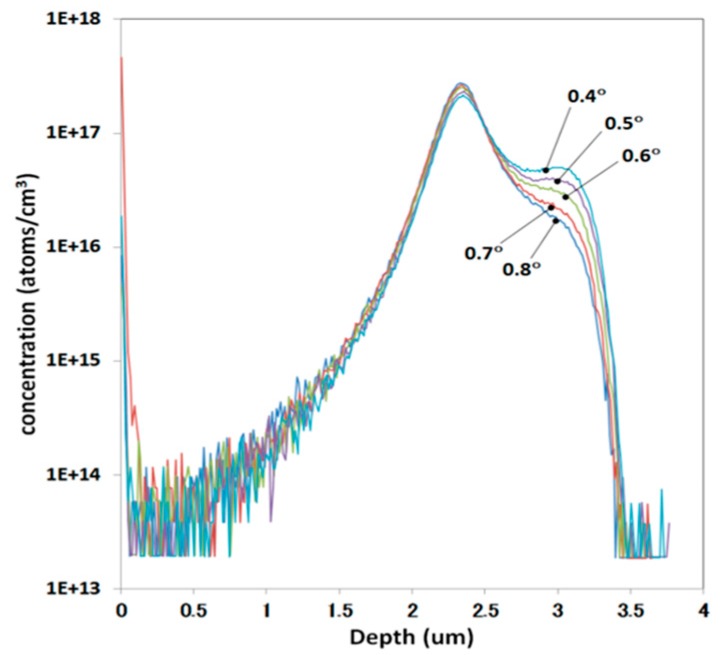
Secondary ion mass spectroscopy (SIMS) profiles implanted in 0.1° tilt steps [31]. Ion: B^+^; Energy: 1.5 MeV; Dose: 1 × 10^13^ cm^−2^; Tilt: 0.4–0.8°.

Lastly, discussion is focused on zero-degree tilt implantation, which is applied to avoid shadowing occurring due to resist patterns and to obtain deeper profiles by intentional channeling. Figure 19 shows the SIMS profiles implanted with 0.4–0.8° tilts in 0.1° steps, 1.5 MeV energy, 1 × 10^13^ cm^−2^ dose of B^+^ [31]. There are clear differences in the SIMS profile even only 0.1° tilt steps due to the channeling differences. Because wafers usually have a small-angle off-angle to obtain good quality epitaxial growth, and the wafer orientation and implanter angle setting contain errors, it is difficult to control channeling at present. Therefore, it can be said that the zero-degree tilt implantation is quite a variable process.

## 6. B vs. BF_2_ for Pinned Photodiode (PPD) Formation

One of the critical implantations is that to form the P^+^ pinning layer of PPD, which affects both the complete signal electron transfer from PD to FD through TG and the dark current/blemishes. If the P^+^ pinning layer is thicker, the signal electron transfer becomes difficult. Its edge position with reference to the TG gate edge is also a sensitive parameter. To reduce dark current and blemishes, a metal-free unit is required because the electric field between the P^+^ pinning layer and N PD is large, as shown in Figure 3.

There are two options for this implantation, i.e., low-energy B^+^ or BF_2_^+^ implantations. Table 2 shows the comparison between them. High-current low energy implanters are preferable for the low-energy and medium-dose B^+^ implantation, such as the SHX series of SMIT, which can provide 200 eV as the minimum energy [17]. Then, the productivity is same even in case of the strong deceleration mode. Doping profiles, including the depth and lateral spread of the two conditions are almost in the same levels. The low-energy B^+^ generates smaller damage because a mass of ^11^B^+^ is 11/49 times smaller than that of ^11^B^19^F_2_^+^. Metal contamination of the low-energy B^+^ is also lower thanks to a smaller knock-on and sputtering effect and less energetic metallic ions, as explained in Section 3.4. Fluorine from BF_2_ has a positive effect for dark current reduction on a case-by-case basis because fluorine can terminate the dangling bonds. In the case of low-energy B^+^, F^+^ can be implanted separately if necessary.

Since the formation of the NPD and the P^+^ pinning layer are complicated in practice, the selection is not straightforward. Simply speaking, the low-energy B^+^ looks better.

## 7. Conclusions

Ion implantation is an indispensable technology for image sensors, and image sensors play a significant role for implantation technology development.

Image sensors are so sensitive to metal contamination that even a single metal atom per pixel can be detected as a blemish. Image sensors have always required metal contamination reduction of ion implanters. The plasma shower using RF plasma generation is a representative example. Although some metal ions, such as ^184^W^19^F^++^ [20] and ^184^W^12^C^+^, cannot be removed by the mass analyzing magnet due to the charge exchange process, the electrostatic angular energy filter after the mass analyzing magnet is a highly effective method to remove such contamination caused by the charge exchange process. The protection layer on the silicon is needed to protect the silicon wafer against the physisorbed metals. The thickness of the protection layer should be determined by considering the knock-on depth. In addition, the wafers should be cleaned up just after ion implantations and before thermal treatments.

Crystal damage by ion implantation also causes blemishes. The damage becomes larger under the following conditions if the other conditions are the same: (a) higher energy; (b) larger dose; (c) higher beam current; (d) longer ion beam irradiation time; (e) shorter ion beam irradiation interval; (f) larger ion mass.

To obtain precise doping profiles, channeling should be reduced. The most effective method is to choose proper tilt and twist angles. If ion implantation direction has some angle to TG to suppress the channeling, for example, multi-step implantation should be applied to keep the symmetry. The screen oxide method is not effective because it needs thick oxide layers. Although the pre-amorphization method is good for channeling suppression, re-crystallization quality is not yet sufficient at present. The zero-degree tilt implantation has large variation because the channeling is sensitive to even small angle variation, especially in a high-energy case.

For P^+^ pinning layer formation, the low-energy B^+^ implantation method might have less metal contamination and damage, compared with the BF_2_^+^ method.

There remain important topics on ion implantation relating to image sensor fabrication, which are not discussed in this paper, such as annealing, high-energy implantation, trench implantation, uniformity, and so on.

## Figures and Tables

**Table 1:** The amorphous layer thickness by As dimer implantation, compared with that by As monomer implantation [22]. The figure illustrates As monomer and dimmer.

Ion	Energy	Dose	Amorphous Thickness
	(keV)	(cm^−2^)	(nm)
As^+^	15	3 × 10^14^	25.5
			∧
As_2_^+^	30	1.5 × 10^14^	26.4
As^+^	15	1.0 × 10^14^	21.3
			∧
As_2_^+^	30	5.0 × 10^13^	22.2
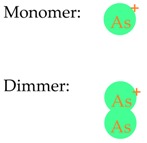			

**Table 2:** Comparison between B^+^ and BF_2_^+^ implantations for forming P^+^ pinning layer at pinned photodiode (PPD).

	Low Energy B^+ (1)^	BF_2_^+^
Productivity	Same	
Shallow Depth	Same	
Lateral Spread	Same	
Damage	Advantage ^(2)^	
Metal Contamination	Advantage ^(3)^	
Fluorine Effect	Separate F^+^ Implantation ^(4)^	Advantage in Some Cases

Note: (1) High-current low energy implanters are assumed, such as SHX series of Sumitomo Heavy Industries Ion Technology (SMIT), which has 200 eV minimum energy [17]; (2) knock-on effect is smaller because the mass of ^11^B^+^ is 11/49 times smaller than that of ^11^B^19^F_2_^+^; (3) smaller sputtering effect and energetic metal contamination; (4) F^+^ can be implanted separately if needed.
